# Determinants of Low Birth Weight in Malawi: Bayesian Geo-Additive Modelling

**DOI:** 10.1371/journal.pone.0130057

**Published:** 2015-06-26

**Authors:** Alfred Ngwira, Christopher C. Stanley

**Affiliations:** 1 Lilongwe University of Agriculture and Natural Resources, Department of Basic Sciences, Lilongwe, Malawi; 2 University of North Carolina Project, Kamuzu Central Hospital, Lilongwe, Malawi; Johns Hopkins Bloomberg School of Public Health, UNITED STATES

## Abstract

Studies on factors of low birth weight in Malawi have neglected the flexible approach of using smooth functions for some covariates in models. Such flexible approach reveals detailed relationship of covariates with the response. The study aimed at investigating risk factors of low birth weight in Malawi by assuming a flexible approach for continuous covariates and geographical random effect. A Bayesian geo-additive model for birth weight in kilograms and size of the child at birth (less than average or average and higher) with district as a spatial effect using the 2010 Malawi demographic and health survey data was adopted. A Gaussian model for birth weight in kilograms and a binary logistic model for the binary outcome (size of child at birth) were fitted. Continuous covariates were modelled by the penalized (p) splines and spatial effects were smoothed by the two dimensional p-spline. The study found that child birth order, mother weight and height are significant predictors of birth weight. Secondary education for mother, birth order categories 2-3 and 4-5, wealth index of richer family and mother height were significant predictors of child size at birth. The area associated with low birth weight was Chitipa and areas with increased risk to less than average size at birth were Chitipa and Mchinji. The study found support for the flexible modelling of some covariates that clearly have nonlinear influences. Nevertheless there is no strong support for inclusion of geographical spatial analysis. The spatial patterns though point to the influence of omitted variables with some spatial structure or possibly epidemiological processes that account for this spatial structure and the maps generated could be used for targeting development efforts at a glance.

## Introduction

Low birth weight (LBW) has been defined by World Health Organization [1] as weight less than 2.5kg. LBW is a leading cause of prenatal and neonatal deaths as such it is a world wide issue and one of the most important public health problems. According to UNICEF and WHO [2], half of low birth weight children are in the South Central Asia where more than a quarter of all born children are less than 2.5kg, representing 27% of all new births with LBW. Sub-Saharan Africa has the second highest incidence of LBW, pegged at 15%. Malawi is part of sub-Saharan Africa with the latest LBW incidence at 12% [3].

Epidemiology of LBW shows that it is multi-factorial. The primary cause of LBW is preterm delivery, occurring less than 37 weeks gestation, and intra uterine growth retardation or association of these two. Other determinants of LBW are smoking [4], low maternal education [5], younger maternal age [6], marital status, slight weight gain during pregnancy, hypertension, genitourinary tract infection in pregnancy, parity and fewer prenatal consultations [7]. In addition, low income family, demographic and reproductive variables such as other children with low birth weight and history of miscarriage have been reported to be associated with low birth weight [8]. Finally, environmental factors such as maternal exposure to air pollutants are also associated with this condition [9]. Furthermore, the risk factors of child birth weight are known to be hierarchical occurring at both the individual-level and the neighborhood-level with complex interactions [10,11]. Most studies have focused on individual level factors of low birth weight. A few studies though have considered the hierarchical nature of child birth weight factors [11–14] but none in Malawi. Our study aimed at investigating factors of low birth weight in Malawi by taking into account the hierarchical nature of child birth weight factors using a Bayesian hierarchical model. The Bayesian hierarchical model modelled second level unit as correlated random component which was the surrogate of unobserved contextual factors. The study assumed the flexible approach of using spline functions for metrical continuous covariates and the geographical random component so as to have detailed relationships between the covariates and the response.

## Materials and Methods

### Study area and data

The study focused on under five children in Malawi and used the standard and nationally representative 2010 Malawi demographic and health survey (MDHS) data. The 2010 MDHS was conducted from June to November in 2010. The 2010 MDHS data was downloaded from the DHS web site after being granted permission. The MDHS was a two stage cluster sampling design with enumeration areas (EAs) as primary sampling units and households as secondary sampling units. EAs were stratified as rural or urban. A total of 849 EAs were sampled with 158 in urban areas and 691 in rural areas. A representative total sample of 27307 households was selected and 25311 households were occupied in the 2010 MDHS. Data collection was by questionnaires. There were three types of questionnaires, women, men and household questionnaire. Households that were successfully interviewed were 24825, yielding a response rate of 98%. Eligible women were 23748 and 23020 were successfully interviewed, yielding a response rate of 97%. Eligible men were 7783 and those that were successfully interviewed were 7175, yielding a response rate of 92%. The data set that was used in the analysis was child record data set which was based on women and household questionnaire.

Data sets were extracted and new variables generated using STATA version 12. Data variables used in this study were based on the variables used in previous studies on child birth weight [4–11]. The response variable in the first extracted data set was child birth weight in kilograms. The covariates in this data set were mother smoking status, mother age in years, mother education, mother height (<150cm, > = 150cm), mother weight (<45kg, 45–70kg, >70kg), number of antenatal visits for pregnancy, birth order (1, 2–3,4–5,6+), wealth index and district of the child. Birth order, mother smoking status, mother education, wealth index, mother height, mother weight and district of the child were categorical variables. All children records where birth weight was missing were dropped so that the final sample size of children for the birth weight data was 13087. The response variable in the second extracted data set was child size at birth which had two categories (smaller than average and average or higher). Child size at birth had actually three categories based on mother recall of child size at birth (very small, smaller than average, average and higher) but two categories were considered so as to have a binary logistic model. Child size at birth was also considered as the response so as to compare results with those where birth weight in kilograms was the response. The covariates in this data set were all those that were in the first data set. Similarly all children records where child size at birth was missing were dropped so that the final sample size of children for the child size at birth data was 19486. The missing covariate values in both data sets were left unremoved.

### Statistical analysis

First univariate logistic regression was performed in STATA version 12 to select potential covariates for the multiple regression models. Covariates that were significant at 20% significance level were considered as candidate variables for multiple regression models. The significance level of 20% rather than 5% was used in selecting covariates for multiple regression analysis so as to allow more potential covariates to be selected. Cross tabulations between categorical covariates and categorized birth weight (< 2.5kg or > = 2.5kg) and child size at birth was done to have percentage distributions of low birth weight and size at birth per covariate categories. The histogram of birth weight in kilograms was also plotted to see the plausibility of Gaussian model.

The following multiple variable hierarchical model was then fitted in R

$$\text{g}(\mu )=X\beta +u_{i}$$ (1)

where g is the link function linking the mean of the response to the predictor *Xβ* + *u*_*i*_, and *u*_*i*_ is the area level random effect representing unmeasured contextual factors. In case of child birth weight as a response, the link function was the identity link resulting in the Gaussian regression model. For the child size at birth as the response, the link function was the log of odds of less than average size at birth resulting in the binary logistic model. To take a more flexible approach, the continuous covariates and the area level random effects were modelled by the nonlinear smooth functions. This revealed their subtle influences which could not be shown if modelled parametrically. To reflect this flexible approach, (1) is changed toa geo-additive model

$$\text{g}(\mu )=w_{i}^{T}\gamma +f_{1}(x_{i1})+f_{2}(x_{i2})+\ldots +f_{p}(x_{ip})+f_{spat}(s_{i})$$ (2)

Where *f*_*j*_ for *j* = 1, 2, 3, …,*p* are smooth functions expressing nonlinear relationship between the response variable and the continuous covariate and *f*_*spat*_(*s*_*i*_) is the area of the child random effect. The vector of coefficients γ determine the parametric relationship between the response and the categorical covariates. The smooth functions *f*_*j*_ were specified as Bayesian splines. According to [15], this assumes approximating *f*_*j*_ by the polynomial spline of degree *l* defined at equally spaced knots $x_{j}^{min}=\zeta_{j0},\zeta_{j1},\ldots \;,\zeta_{js}=x_{j}^{max}$ which are within the domain of the covariate *x*_*j*_. The Bayesian spline can be written as a linear combination of *d* = *s*+*l* basis functions, *B*_*m*_, that is,

$$f_{j}\left(x_{j}\right)=\sum_{m=1}^{d}\varepsilon_{jm}B_{m}\left(x_{j}\right)$$ (3)

Now Bayesian estimation of the penalized spline (3) is equivalent in estimating model parameters *ε*_*j*_ = (*ε*_j1_,*ε*_j2_,…,*ε*_jm_) where first or second order random walk priors for the regression coefficients are assigned. A first order random walk prior for equidistant knots is given by: *ε*_*jm*_ = *ε*_*j*,*m-1*_ + *u*_*j*.*m*_ where *m* = 2,3,…,*d* and a second order random walk prior for equidistant knots is given by: *ε*_*jm*_ = 2*ε*_j,m-1_+*ε*_j,m-2_+*u*_*j*.*m*_ where *m* = 3,4,…,*d* and $u_{j.m}\sim N(0,\tau_{j}^{2})$ are random errors. The spatial effect was modelled by the tensor product of two dimensional p-spline defined as

$$f_{spat}\left(x_{1},x_{2}\right)=\sum_{i}^{k}\sum_{j}^{k}B_{spat,\;ij}B_{1i}\left(x_{1}\right)B_{2j}\left(x_{2}\right)$$ (4)

where (*x*_1_,*x*_2_) refers to the coordinates of the location of the data point, latitude and longitude, or location centroids based on the map. Note that *f*_*spat*_(*x*_1_,*x*_2_) represents the effect of correlated unmeasured or unobserved location effects. The prior for *B*_*spat*,*ij*_ = (*B*_spat,11_,*B*_spat,12_,…,*B*_*spat*,*kk*_) is based on spatial smoothness priors common in spatial statistics [16]. The most commonly used prior specification based on the four nearest neighbours is defined as:

$$B_{spat,\;ij}|.\sim N(B_{spat,i-1\;j}+B_{spat,i+1\;,j}+B_{spat,i,j-1}+B_{spat,i,j+1},\frac{\tau_{ij}^{2}}{4})$$

For *i*,*j* = 2,…,*k*-1 with appropriate changes for corners and edges. Since model estimation was by empirical Bayesian method, all variance parameters were treated as unknown constants that were estimated by restricted maximum likelihood (REML) method and hence their priors were not given. The fixed effects were assigned diffuse priors. An advantage of the empirical Bayesian inference over full Bayesian inference is that questions about the convergence of Markov Chain Monte Carlo (MCMC) samples or sensitivity on hyper parameters do not arise [17]. Further more, a comparison of full Bayesian and empirical Bayesian approach in a simulation study, has shown empirical Bayesian approach yielding better point estimates, especially for Bernoulli distributed responses [18].

## Results

### Descriptive summaries

The percentage of low birth infants is higher in young mothers (aged 20 years or less) and in older mothers (aged 35–49 years) than in mothers aged 20–34 years (Table 1). By birth order, the percentage of low birth weight infants is higher for first births than for the subsequent births. There is an inverse relationship between low birth weight and mother education. The same trend is observed among wealth quintile. As education and household wealth increase, the percentage of low birth infants decrease. For example the percentage of low birth weight decreases from 13 percent among mothers with no education to 7 percent among mothers with more than secondary education. Likewise percentage of births in which infants weigh less than 2.5kg decreases from 14 percent among mothers in the lowest wealth quintile to 11 percent among mothers in the highest quintile. Among the regions (Table 2), the southern region has the smallest proportion of low birth weight infants and the central region has the highest (11 and 14 percent respectively). Similar patterns in education and wealth quintile are seen for births categorized as very small and smaller than average as was seen for births less than 2.5kg.

**Table 1:** Percentage distribution of birth weight and size at birth for some covariates.

Variable	Birth weight	Size of child at birth	
	less than 2.5kg	very small	less than average
**Mother age at birth**			
<20	15.4	4.6	14.4
20–34	11.2	3.7	10.8
35–49	14.5	5.3	10.9
**Birth order**			
1	15.0	4.6	14.4
2–3	11.0	3.3	11.1
4–5	11.0	4.4	10.1
6+	13.3	4.2	10.9
**Mother smoking**			
Smoke tobacco	14.0	10.4	15.4
Does not	12.3	4.0	11.5
**Mother education**			
No education	13.3	4.7	12.9
Primary	12.8	4.1	11.8
Secondary	10.2	3.0	8.7
Higher	7.0	2.9	5.5
**Wealth index**			
Poorest	13.5	4.7	12.8
Poor	13.2	4.0	12.7
Rich	12.6	4.4	10.6
Richer	11.8	3.6	11.0
Richest	10.6	3.2	9.9

**Table 2:** District percentage distribution of birth weight and size at birth.

District	Birth weight	Size of child at birth	
	less than 2.5kg	very small	less than average
**Northern Region**	11.6	5.2	9.7
Chitipa	9.6	7.2	10.3
Karonga	8.9	4.6	8.8
Nkhata-bay	9.6	4.8	6.4
Rumphi	9.5	5.3	9.6
Mzimba	13.6	5.2	10.6
**Central Region**	13.5	3.9	11.3
Kasungu	11.9	3.8	8.8
Nkhota-kota	11.3	3.7	12.5
Ntchisi	12.3	4.0	6.5
Dowa	13.1	4.5	8.5
Salima	11.0	4.0	7.2
Lilongwe	17.2	3.5	9.7
Mchinji	14.8	4.1	28.0
Dedza	13.0	6.4	11.9
Ntcheu	9.2	0.9	10.6
**Southern Region**	11.3	3.8	12.1
Mangochi	9.4	2.1	20.0
Machinga	9.5	5.2	11.3
Zomba	10.1	3.8	13.9
Chiradzulu	12.2	4.4	7.3
Blantyre	12.6	4.3	9.8
Mwanza	9.3	2.1	6.7
Thyolo	16.7	4.2	17.5
Mulanje	11.1	3.0	8.4
Phalombe	9.9	5.0	7.1
Chikhwawa	10.5	5.6	9.9
Nsanje	7.5	2.7	6.2
Balaka	11.0	2.8	8.8
Neno	11.6	3.7	10.1

**Fig 1:**
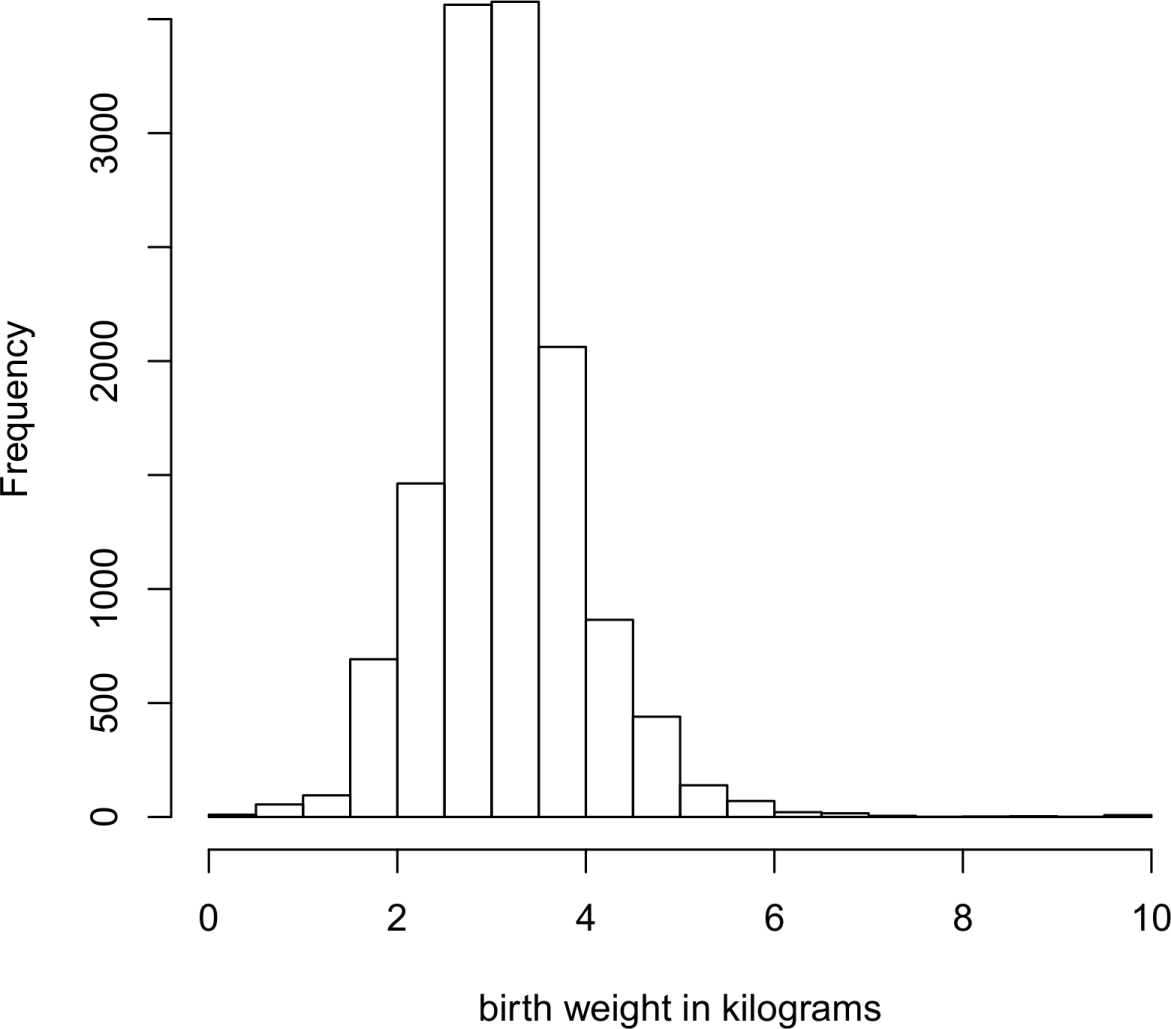
Histogram of child birth weight in kilograms

To check the suitability of a Gaussian model, a histogram of birth weight was plotted. Fig 1 gives the histogram of birth weight in kilograms. The histogram shows that birth weight is symmetrically distributed. This leads to a simpler model and analysis since the Gaussian assumption is more tenable.

### Empirical Bayesian

#### Fixed effects

Fixed variables associated with child birth weight under the Gaussian model are birth order, mother weight, and mother height (Table 3). The birth order effects are positive which means children with higher birth order are associated with higher child birth weight than children of lower birth order. Positive effect of mother weight means that as mother weight increase child birth weight also increase. The positive effect for mother height means that the taller the mother the higher the child birth weight as well. Factors associated with child size at birth under binary logistic model are birth order 2–3 and 4–5, wealth index of richer family, mother education of secondary category, and mother height (Table 3). The effects of birth order 2–3 and 4–5 are negative which means children of birth order 2–3 and 4–5 are associated with lower risk of being small at birth compared to those with first birth order. Mother secondary education has a negative effect on child size at birth which means children of secondary education mothers have reduced chance of being small at birth than children of mothers with no education. Negative effect of mother height on child birth size means that children of mothers whose height is equal to or greater than 150 cm are less likely to be smaller than average size at birth than children of mothers whose height is less than 150 cm.

**Table 3:** Summary of Gaussian and binary logistic models.

Variable	Gaussian	Logistic
	Coefficient(95%CI)	Coefficient(95%CI)
**Intercept**	2.851(2.645 3.058)	-0.482(-1.003 0.039)
**Birth order**		
1	_	_
2–3	0.105*(0.014 0.196)	-0.350*(-0.657–0.043)
4–5	0.138*(0.017 0.258)	-0.540*(-0.952–0.129)
6+	0.166*(0.018 0.314)	-0.406(-0.902 0.090)
**Wealth index**		
Poorest	_	_
Poor	0.058(-0.029 0.146)	-0.151(-0.414 0.111)
Rich	0.027(-0.058 0.112)	-0.225(-0.493 0.044)
Richer	0.047(-0.040 0.134)	-0.340*(-0.631–0.048)
Richest	0.056(-0.039 0.152)	-0.302(-0.643 0.040)
**Weight**		
<45kg	_	_
45–70kg	0.163*(0.064 0.263)	-0.262(-0.548 0.024)
>70kg	0.214*(0.065 0.363)	-0.262(-0.775 0.251)
**Height**		
<150cm	_	_
≥150cm	0.126* (0.044 0.208)	-0.500*(-0.740–0.260)
**Mother education**		
No education	_	_
Primary	-0.004(-0.090 0.082)	-0.200(-0.450 0.049)
Secondary	-0.020(-0.131 0.091)	-0.398*(-0.779–0.017)
Higher	-0.160(-0.467 0.147)	-0.702(-2.195 0.792)
**Variance Components**		
**Spatial effect**	0.0227	0.6396
**Non-linear effects**		
Prenatal visits	0.0000	0.0016
Mother age	0.0003	0.0017

^_^ means reference category, and

* means significant at 5% significance level

#### Nonlinear effects

**Fig 2:**
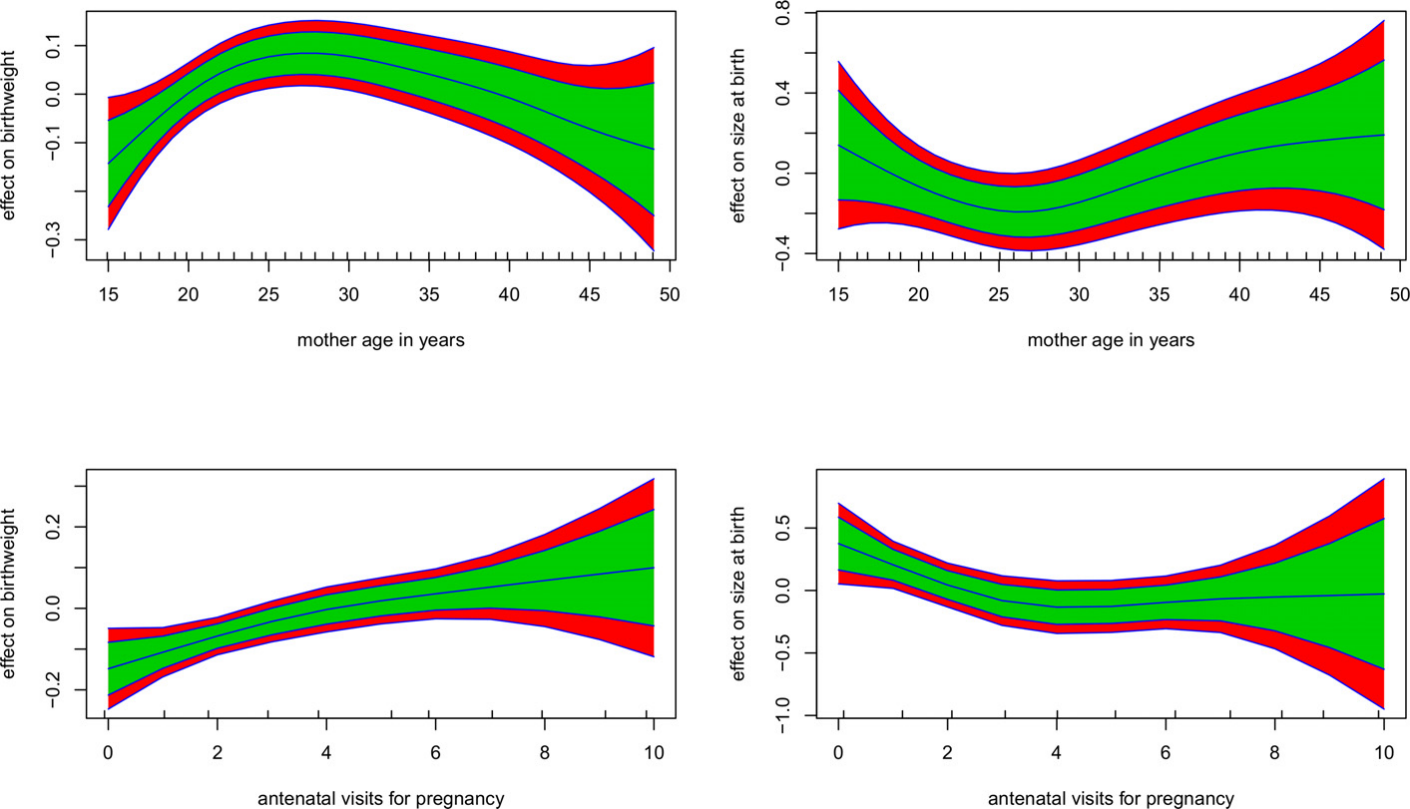
Nonlinear terms from the Gaussian and logistic model (left and right). Red band (95% CI) and green band (80% CI).

Starting with the nonlinear effects to child birth weight (Fig 2 left), children of young mothers (aged 15 to 23 years) and older mothers (aged 35 to 49 years) are more likely to have low birth weight than children of mothers aged 23 to 35 years. Furthermore as number of antenatal visits for pregnancy increase, child birth weight also increases. With regard to nonlinear effects to child size at birth (Fig 2 right), children of mothers aged 15 to 25 years and children of mothers aged 35 to 49 years are prone to have small size at birth than children of mothers aged 25 to 35 years. Children whose mothers have less prenatal visits are prone to be small at birth.

#### Spatial effects

**Fig 3:**
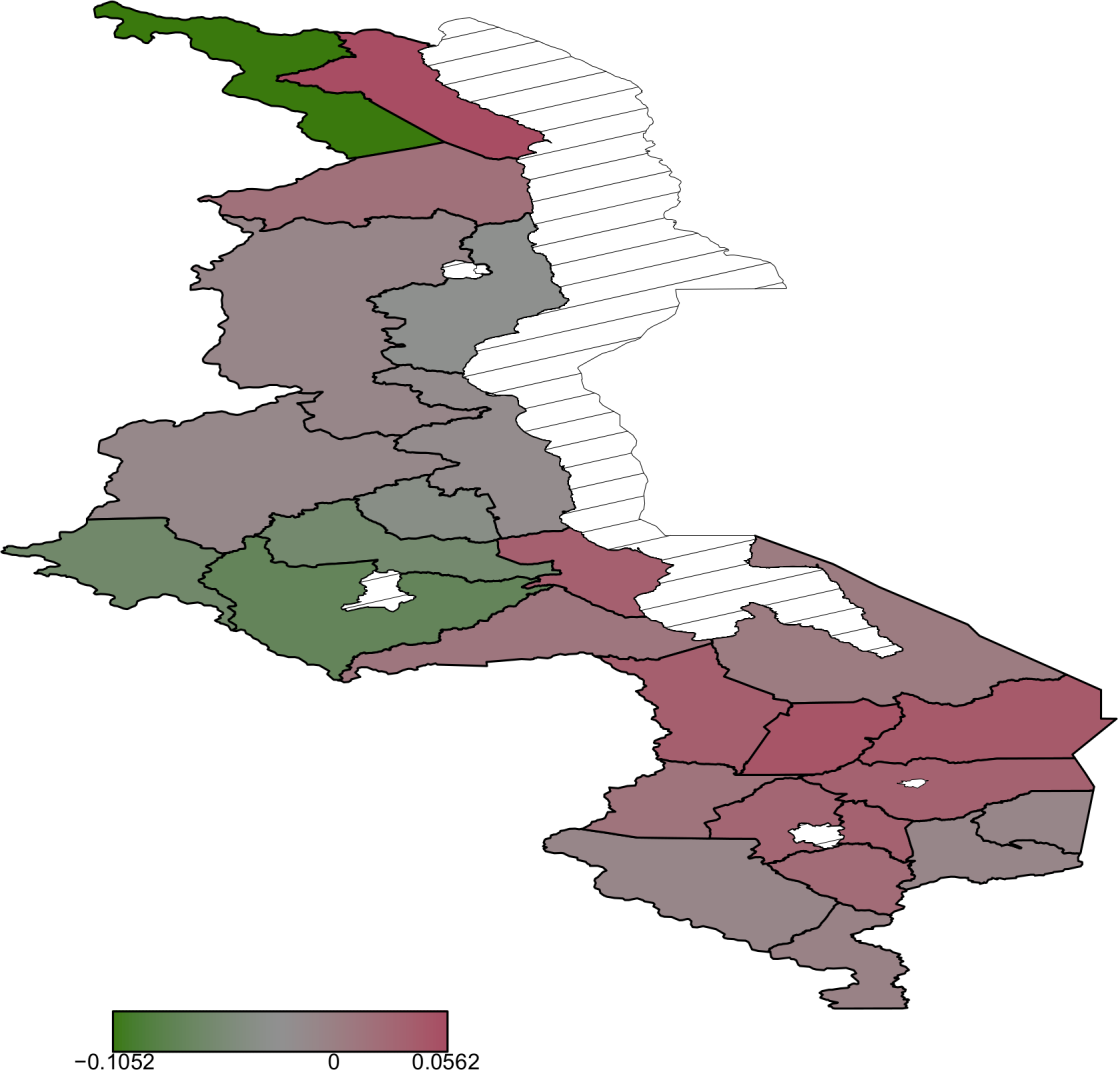
Residual spatial patterns from the Gaussian model .

**Fig 4:**
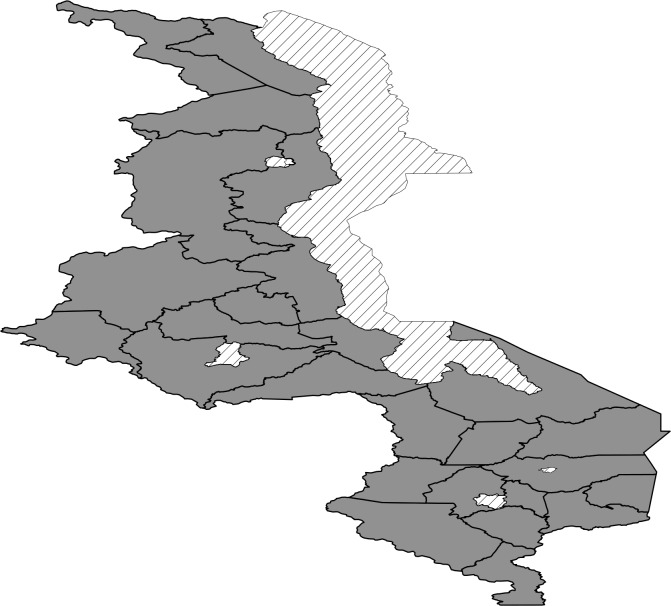
The 95% posterior credible intervals map for the spatial effects from the Gaussian model. Red means positive effect, green means negative effect and grey means insignificant effect.

**Fig 5:**
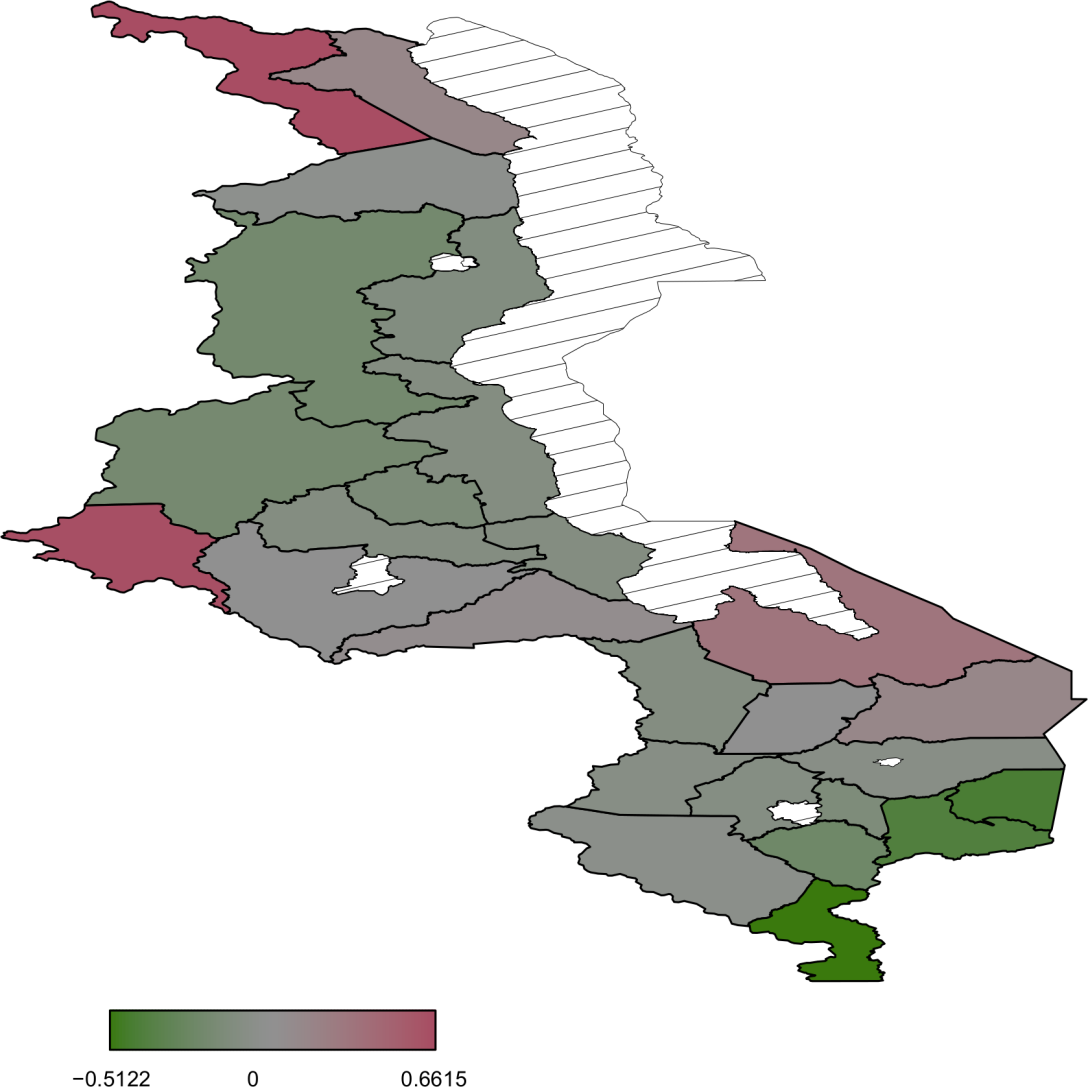
Residual spatial patterns from the logistic model.

**Fig 6:**
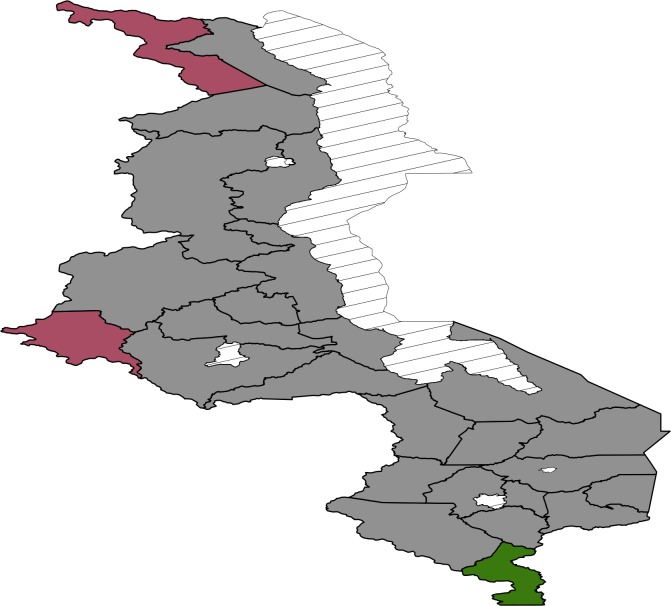
The 95% posterior credible intervals map for the spatial effects from the logistic model. Red means positive effect, green means negative effect and grey means insignificant effect.

Most areas in the south are associated with increased birth weight (Fig 3) while north and central regions have a mixture of areas increasing birth weight and decreasing birth weight. Posterior probability map thoughindicates that there is no significant variation in the residual spatial effects to birth weight (Fig 4). With regard to residual spatial effects to child size at birth (Fig 5), Chitipa, Mchinji and Mangochi are associated with increasedrisk of child being small at birth while Phalombe, Mulanje and Nsanje decrease the risk of child being small at birth. Areas showing significant spatial effects to child size at birththough are Chitipa, Mchinji and Nsanje (Fig 6).

## Discussion

This study found that child birth order, mother weight, mother height, mother education and family wealth are significant predictors of birth weight. The findings generally confirm what is in the literature. The positive effect of birth order on birth weight in this study is in consistent with that of [19] which found birth order as an important factor influencing birth weight and that first order births are on average more likely to be small babies than higher order births. The finding of a positive effect of mother weight, and height on birth weight is also in line with [20] where it is said that such a relationship is because mother weight and height reflect food taken which has a direct influence on child birth weight. The mechanisms associated with small size at birth among the less educated according to [5] may include poor diet as a result of low dietary literacy. Furthermore limited education may also result in limited access to prenatal care, especially in settings where clients are expected to pay for service. Positive effect of family wealth on child birth weight may be due to the fact that wealth is associated with income level which determines kind of diet.

The study has also documented that women at the reproductive ages of 25 years or less and 35 years and over are more prone to deliver low birth weight or small sized babies (Fig 2 top left and Fig 2 top right). Mothers less than 25 years are actually prone to have physical and emotional maturity issues which may contribute to their elevated incidence of small size births or low birth weight infants. Their ignorance of how to take care of themselves during pregnancy works against child birth weight or size at birth. Accordingly, among mothers who are 35 years or older, there is a greater tendency to develop prenatal complications and a higher probability of inadequate nutrition, thus increasing their likelihood of delivering less than average size or low birth weight babies. The study has further shown that mothers whose prenatal visits are less than four are prone to have low birth weight or less than average size babies (Fig 2 bottom left and Fig 2 bottom right). Increased prenatal visits ensure mothers receive adequate diet literacy which helps improve child birth weight.

The observed residual spatial patterns in child birth weight and child size at birth may be due to unobserved factors not captured by the covariates in the models, and it is a matter of conjecture to identify them. According to [21], some of the possible factors are the area natural resources such as soil type and land slope, area population density, and distance to health facilities. Natural resources like soil type and slope may have an impact on crop yield which may affect mother nutrition. Population density may affect spatial variation in child birth weight or child size at birth in the way that, high population density may result in competition for food in area which may affect mother nutrition status. Mother nutrition status in turn may have a direct effect on child birth weight. Distance to health facility affect mother frequency of going to the health facilities for prenatal care which has an influence on child birth weight.

The study was not without weaknesses. Due to the cross-sectional nature of the data collection exercise, no temporal linkages can be made between birth weight or size at birth and any of the explanatory variables. Moreover, because the analysis was based on an existing data set, the study was limited to the use of variables found in the data set. For instance, our study did not take into account the effect of histories of maternal health and pregnancy (previous abortion/miscarriage) which was found to be significantly associated with the incidence of low birth weight by [22].

## Conclusion

The study found support for the flexible modeling of some covariates that clearly have non-linear influences. Nevertheless there is no strong support for inclusion of geographical spatial analysis as there was no significant spatial variation of child birth weight under Gaussian modelling and that most areas showed insignificant spatial effects to child size at birth under binary logistic model The spatial patterns shown though point to the influence of omitted variables with some spatial structure or possibly epidemiological processes that account for this spatial structure, and the maps generated could be used for targeting development efforts at a glance.

## Supporting Information

S1 File Gaussian model data (CSV)

S2 File Binary logistic model data (CSV)

S3 File Map of Malawi file (CSV)
